# Evaluating the efficacy of frenotomy in breastfeeding success: a review of current guidelines and outcomes

**DOI:** 10.3389/fped.2025.1702138

**Published:** 2025-12-08

**Authors:** Kennedy A. Sabharwal, Michael W. Simon

**Affiliations:** 1 College of Medicine, University of Kentucky, Lexington, KY, United States; 2 Department of Pediatrics, University of Kentucky, Lexington, KY, United States

**Keywords:** ankyloglossia, tongue-tie, frenotomy, breastfeeding, frenectomy, lactation

A new mom struggles with breastfeeding. Painful latching leads to Google, then tongue-tie Facebook groups where surgery seems like the obvious answer. Within days, she is scheduled for frenotomy with minimal evaluation of positioning, latch issues, or other common breastfeeding problems. This happens constantly now. Between 1997 and 2012, ankyloglossia diagnoses jumped nearly ten-fold along with frenotomy procedures (1). What used to be managed with positioning help and lactation support now defaults to surgery.

We have expanded surgical indications beyond tongue frenotomy to lip and cheek procedures, despite weak evidence. Traditional lactation support gets skipped for immediate surgical referrals. The real question is not whether frenotomy helps short-term, it is whether we are massively overtreating something that usually resolves with good support.

The medical consensus is clear. The 2020 Academy of Otolaryngology review found unclear associations between breastfeeding problems and lip ties, recommending non-medical management first (2). The AAP agrees, lactation support must be pursued before surgery (3). The data on frenotomy outcomes is mixed at best. Some studies show short-term improvements, but exclusive breastfeeding rates drop significantly between 2 weeks and 2 months post-procedure (4). Even studies showing benefits have wide variability and inconsistent outcome measures.

However, we must examine the studies that also advocate for frenotomy. Several randomized controlled trials have documented meaningful maternal improvements following frenotomy. One study found no objective improvement in breastfeeding success at 5 days or 8 weeks, but mothers reported significantly higher breastfeeding confidence and fewer switched to bottle feeding early (5). Earlier papers demonstrated reduced maternal nipple pain and improved infant latch scores, while another publication documented similar immediate pain relief (6, 7).

These positive findings represent real clinical benefit that can impact breastfeeding success and family wellbeing; however, few studies have examined whether symptom improvements translate into better long-term breastfeeding outcomes.

The positive literature suggests a subset of mothers and infants experience genuine benefit from frenotomy, particularly those with documented functional restriction and significant feeding difficulties despite adequate lactation support (8). Rather than dismissing this evidence, we believe the best approach involves using these findings to guide individualized decision-making.

Although there is insufficient evidence for lip and cheek frenectomy, these procedures are everywhere (2). Surgical indications have outpaced actual evidence. A study of 115 infants referred for tongue-tie surgery found 63% did not need the procedure (9). That is not statistical noise, that is systematic overdiagnosis.

The disparities tell the whole story. Kids with ankyloglossia diagnoses are more likely from higher-income areas, have private insurance, and live in the Midwest (1). Access to specialists, not medical necessity, drives who gets treated. Social media makes it worse. Tongue-tie awareness campaigns increase diagnoses and procedures (3). Parents show up expecting surgery instead of evaluation. Without standardized criteria or training requirements, providers make wildly different decisions for identical cases.

We do not know the long-term effects of early surgical intervention. Emerging evidence suggests risks like oral defensiveness, sensory feeding issues, and food restrictions (3). This matters because most tongue-tied babies end up breastfeeding fine without surgery. Good lactation support beats surgery for long-term success. Comprehensive approaches addressing positioning and ongoing guidance provide sustained benefits that surgery alone cannot match.

We must start with comprehensive lactation assessment, positioning help, and intensive support before even thinking about surgery. This matches professional guidelines and avoids potential complications. Providers need better training in ankyloglossia assessment and standardized criteria. Institutions need protocols that prioritize conservative management with clear thresholds for surgery based on actual functional problems, not just having a tongue-tie.

Parent education is huge. We need to counter social media misinformation by explaining that most breastfeeding difficulties come from positioning, latch problems, and maternal factors, not anatomy requiring surgery. Lactation support, not surgery, should be first-line for tongue-tie management. We need to resist the pressure for quick surgical fixes and actually address the complex reasons breastfeeding can be difficult. Frenotomy might help carefully selected patients with clear functional problems, but routine surgery is not justified by current evidence. The potential for complications plus systematic overdiagnosis demands we get back to evidence-based conservative care.

We need standardized criteria, better training, and patient education that counters social media hype. Most importantly, successful breastfeeding depends more on skilled support than surgical intervention. Our patients deserve evidence-based care, not social media trends.
